# Annual acknowledgement of manuscript reviewers

**DOI:** 10.1186/s12962-016-0054-3

**Published:** 2016-03-11

**Authors:** Joan Rovira

**Affiliations:** Cost Effectiveness and Resource Allocation, University of Barcelona, Barcelona, Spain

## Abstract

The editors of Cost Effectiveness and Resource Allocation would like to thank all our reviewers who have contributed to the journal in volume 13 (2015).

**Fernando Antonanzas**

Spain

**Denizar Araujo**

Brazil

**Mattias Aronsson**

Sweden

**Philip Ayieko**

Kenya

**Amilcar Azamar-Alonso**

Mexico

**Philippe Beutels**

Australia

**Abdesslam Boutayeb**

Morocco

**Natalie Carvalho**

Australia

**Usa Chaikledkaew**

Thailand

**Nathorn Chaiyakunapruk**

Australia

**Susmita Chatterjee**

India

**Vijayalakshmi Chelliah**

UK

**Paolo Cortesi**

Italy

**Alexander Cowell**

USA

**Rafael da Silva Barbosa**

Brazil

**George Dalekos**

Greece

**Victoria de Menil**

UK

**Michael Doumas**

Greece

**Ali Emrouznejad**

UK

**Victoria Fan**

USA

**Ewan Gray**

UK

**Liesl Hagan**

USA

**Hassan Haghparast-Bidgoli**

UK

**Samia Hurst**

Switzerland

**David Hutton**

USA

**Inaki Imaz**

Spain

**Roberto Iunes**

USA

**Kjell Arne Johansson**

Norway

**Mary Justin-Temu**

Tanzania

**Prapan Jutavijittum**

Thailand

**Sun-Young Kim**

USA

**Wantanee Kulpeng**

Thailand

**Carol Levin**

USA

**Sung Lim**

USA

**Arindam Nandi**

USA

**Justice Nonvignon**

Ghana

**Eric Obikeze**

Nigeria

**Ogbonnia Ochonma**

Nigeria

**Zachary Olson**

USA

**Supasit Pannarunothai**

Thailand

**Nikolaos Polyzos**

Greece

**Neha Raykar**

India

**Paul Revill**

UK

**Lora Sabin**

USA

**Thaworn Sakunphanit**

Thailand

**Cesar Sanabria**

Peru

**Annie Simpson**

USA

**Ricardo Tellez-Cabrera**

USA

**Rosana Tsuji**

Brazil

**Maduka Ughasoro**

Nigeria

**Anil Vaidya**

The Netherlands

**Laura Vallejo-Torres**

Spain

**Tazio Vanni**

Brazil

**Patrícia Varela**

Brazil

**Alessandro Vitale**

Italy

**Margarethe E. Wacker**

Germany

**Daniel Wattson**

USA

**Sophie Whyte**

UK

**David Wu**

Australia

